# A human breast cancer model for the study of telomerase inhibitors based on a new biotinylated-primer extension assay

**DOI:** 10.1038/sj.bjc.6690526

**Published:** 1999-07

**Authors:** E Raymond, D Sun, E Izbicka, G Mangold, E Silvas, B Windle, S Sharma, H Soda, R Laurence, K Davidson, D D Von Hoff

**Affiliations:** Human Telomerase Working Group, Institute for Drug Development – Cancer Therapy and Research Center, 14960 Omicron Drive, San Antonio, TX, 78245-3217, USA

**Keywords:** new telomerase assay, chemotherapy, telomerase inhibitors, telomere length, porphyrin, cisplatin

## Abstract

Telomerase is an RNA-dependent polymerase that synthesizes telomeric DNA (TTAGGG)_n_ repeats. The overall goal of our work was to establish human cancer models that can be used to design clinical trials with telomerase inhibitors. The objectives of this study were (1) to set up a human breast cancer system that allows evaluation of the effects of telomerase inhibitors in cultured cells using a non-amplified telomerase assay and (2) to test this system using two drugs (cisplatin and TMPyP_4_) that affect the telomerase expression in breast cancer cells in culture. We first compared the telomerase activity in a variety of human breast cancer cell lines to that of other tumour types using a new biotinylated-primer extension assay. Our method, based on a non-amplified primer extension assay shows the direct incorporation of ^32^P-labelled nucleotides induced by telomerase on human telomeric primers. The ^32^P-dGTP labelled telomerase-extended 5′-biotinylated (TTAGGG)_3_ primer can subsequently be separated using streptavidin-coated magnetic beads. As compared to other non-amplified method, we showed that this procedure improved the characterization and the quantification of the banding pattern resulting from telomerase extension by reducing the radioactive background. Using this method, we observed that telomerase activity varies markedly in a panel of 39 human cancer cell lines. For example, MCF7 breast cancer cells in culture showed intermediate telomerase activity corresponding to 33.8 ± 3.4% of that of the HeLa cells (reference cell line). Similarly, the telomere length varied with each cell line (average: 6.24 ± 6.16). No correlation between the level of telomerase and telomere length was observed, suggesting that a high processivity is not required to maintain telomeres and that, in some cell lines, another mechanism of telomere elongation can maintain telomere length. From this study, we selected MCF7 and MX1 models that showed reproducible telomerase activity and a relatively limited telomere length for the testing of potential telomere–telomerase interacting agents. Using cisplatin and a new porphyrin-derived compound TMPyP_4_, we showed that our model was able to detect a down-regulation of the telomerase activity in MCF7 cells in culture and in a human MX1 tumour xenografts. Based on these results, a breast cancer model for evaluating telomerase and telomere interactive agents is proposed. © 1999 Cancer Research Campaign


*British Journal of Cancer* (1999) **80**(9), 1332–1341
© 1999 Cancer Research Campaign
Article no. bjoc.1999.0526

### A human breast cancer model for the study of telomerase inhibitors based on a new biotinylated-primer extension assay

**E Raymond, D Sun, E Izbicka, G Mangold, E Silvas, B Windle, S Sharma, H Soda, R Laurence, K Davidson and DD Von Hoff**

Human Telomerase Working Group, Institute for Drug Development – Cancer Therapy and Research Center, 14960 Omicron Drive, San Antonio, TX 78245-3217, USA

**Summary** Telomerase is an RNA-dependent polymerase that synthesizes telomeric DNA $(TTAGGG)_n$ repeats. The overall goal of our work was to establish human cancer models that can be used to design clinical trials with telomerase inhibitors. The objectives of this study were (1) to set up a human breast cancer system that allows evaluation of the effects of telomerase inhibitors in cultured cells using a non-amplified telomerase assay and (2) to test this system using two drugs (cisplatin and $TMPyP_4$) that affect the telomerase expression in breast cancer cells in culture. We first compared the telomerase activity in a variety of human breast cancer cell lines to that of other tumour types using a new biotinylated-primer extension assay. Our method, based on a non-amplified primer extension assay shows the direct incorporation of $^{32}P$-labelled nucleotides induced by telomerase on human telomeric primers. The $^{32}P$-dGTP labelled telomerase-extended 5′-biotinylated $(TTAGGG)_3$ primer can subsequently be separated using streptavidin-coated magnetic beads. As compared to other non-amplified method, we showed that this procedure improved the characterization and the quantification of the banding pattern resulting from telomerase extension by reducing the radioactive background. Using this method, we observed that telomerase activity varies markedly in a panel of 39 human cancer cell lines. For example, MCF7 breast cancer cells in culture showed intermediate telomerase activity corresponding to 33.8 ± 3.4% of that of the HeLa cells (reference cell line). Similarly, the telomere length varied with each cell line (average: 6.24 ± 6.16). No correlation between the level of telomerase and telomere length was observed, suggesting that a high processivity is not required to maintain telomeres and that, in some cell lines, another mechanism of telomere elongation can maintain telomere length. From this study, we selected MCF7 and MX1 models that showed reproducible telomerase activity and a relatively limited telomere length for the testing of potential telomere–telomerase interacting agents. Using cisplatin and a new porphyrin-derived compound $TMPyP_4$, we showed that our model was able to detect a down-regulation of the telomerase activity in MCF7 cells in culture and in a human MX1 tumour xenografts. Based on these results, a breast cancer model for evaluating telomerase and telomere interactive agents is proposed.

**Keywords:** new telomerase assay; chemotherapy; telomerase inhibitors; telomere length; porphyrin; cisplatin

Human telomere DNA consists of simple repeated TTAGGG-sequences oriented 5′ to 3′ towards the chromosome termini (Blackburn, 1991). The length of the terminal restriction fragment containing the telomere repeats varies from 5 to 15 Kbp in human somatic cells and decreases during cell senescence and aging (Levy et al, 1992; Allsopp and Harley, 1995; Healy, 1995; Shay et al, 1995*a*). Additional results support the important role of telomeres in chromosome stabilization, attachment to the nuclear matrix, and effects on the expression of adjacent genes in normal cells (Price, 1993; Gilley and Blackburn, 1994; Greider, 1994; Healy, 1995). Convergent evidence leads to the hypothesis that telomeric attrition could act as a cell division counter limiting the proliferative somatic cell life span (Greider, 1993; Harrington et al, 1997; Marcand et al, 1997). Although cultured cancer cells exhibit a relative telomeric attrition, most of them have a seemingly unlimited proliferative lifespan.

*Received 15 May 1998*
*Revised 26 November 1998*
*Accepted 13 January 1999*

*Correspondence to:* E Raymond, Department of Medicine, Institute Gustave-Roussy, 39, rue Camille-Desmoulins, 94805 Villejuif, France

The length of telomeres is maintained over time by telomerase, a ribonucleoprotein containing a short RNA motif serving as a template for synthesizing TTAGGG-sequences (Shay et al, 1994). Telomerase activity can be measured in vitro by a primer extension assay in which telomerase synthesizes telomeric repeats onto oligonucleotide $(TTAGGG)_n$ primers (Shay et al, 1994). Such a conventional telomerase assay was initially used to determine the telomerase activity in cell lines but was not sensitive enough to allow detection of telomerase in tissue samples from patients because of the limited number of cells. Therefore, Kim et al (1994) developed a more sensitive method based on a polymerase chain reaction (PCR) technique. Using this method, designated TRAP-assay, they demonstrated that telomerase is linked to cell immortality, stringently repressed in normal somatic cells and reactivated in cancer. The use of a primer with high affinity for telomerase and PCR-amplification have increased the sensitivity of the TRAP-assay allowing detection of telomerase in samples containing fever than 100 tumour cells. We initially investigated compounds that interfered with either the primer used in the TRAP assay or with the *Taq* polymerase enzyme. This prompted us to develop an assay designed to increase the telomerase signal without the use of PCR-amplification. In previous preliminary biochemical studies, we experimented a new biotinylated-primer extension assay for detecting telomerase activity. We showed that this procedure reduces the radioactive background, improves the sensitivity as compared to other conventional non-PCR methods, and allows a better quantification of the telomerase signal as compared to current conventional techniques (Sun et al, 1996, 1997; Thompson et al, 1996; Wheelhouse et al, 1998).

Advanced breast cancer represents a major challenge for the development of new anticancer drugs (Fisher, 1996). Despite a relatively high sensitivity to chemotherapy, the median duration of survival of patients with metastatic breast cancer remains about 24 months (Piccart et al, 1995). Attempts to increase the dose intensity of chemotherapy led to an increase in response rates but minimal improvement in survival with most of the patients experiencing recurrences within the first year after intensive chemotherapy. It has previously been reported that telomerase is expressed during breast epithelial cell immortalization (Shay et al, 1993*a*, 1995*b*). Subsequent reports have indicated that advanced human breast cancer cells overexpressed telomerase in 95% of cases (Hiyama et al, 1996). In addition, there are data indicating an increase in the telomerase activity between early and late breast cancer stages (Hiyama et al, 1996; Bednarek et al, 1997; Tsao et al, 1997). Therefore, telomerase and telomeres may represent interesting targets for the development of new anticancer agents for patients with advanced breast cancers (Raymond et al, 1996).

To date, there is little information regarding telomerase activity using non-PCR-based techniques and attempting to correlate that activity with telomere length in cell line that may be used for testing new compounds against cultured human breast cancer cells. Therefore, in this study we established the experimental conditions for testing telomerase inhibitors in a human breast cancer model. We first have developed our new biotinylated-primer extension assay to measure telomerase activity in extracts from human breast cancer cell lines and xenografts. Using this method, we have investigated the level of functional telomerase activity and attempted to correlate telomerase activity with telomere lengths in human breast cancer cells. We subsequently evaluated the effects of several antiproliferative agents on telomerase expression in culture, including cisplatin and a porphyrin compound $TMPyP_4$. Our data provide information that might help with the design of preclinical and clinical studies using compounds specifically designed to target telomerase and telomeres in human breast cancer cells.

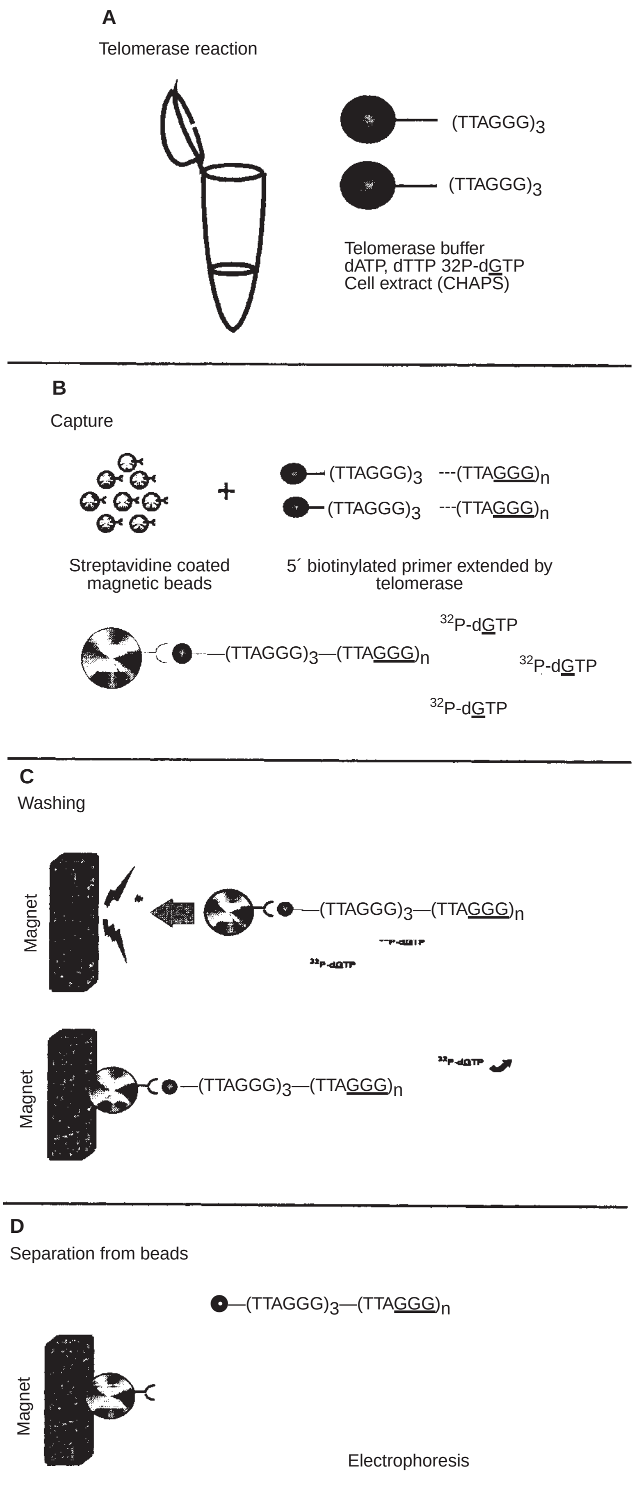

**Figure 1** Biotinylated-primer extension telomerase assay. Telomerase reaction was performed in the presence of biotinylated-$(TTAGGG)_3$ primers (**A**). Biotinylated primers extended by telomerase were captured by streptavidin-coated magnetic beads (**B**). The extended primers attached to the beads were separated and washed to remove free $^{32}$P-dGTP (**C**). The extended primers were separated from the beads and then analysed by electrophoresis (**D**)

### MATERIALS AND METHODS

### Cell collections

Human cervix (HeLa), breast (oestrogen-sensitive MCF71, oestrogen-resistant MCF7m, anthracyclin-resistant MCF7mdr, p53 mutated MCF7-VHK4, MCF7-V4B, T47D, ZR75-1, BT20, HS578t, HS578bst, MDA-MB-231, MDA-MB-330, MDA-MB-435); prostate (LNCaP, DU145, TSUPr1, PC3); ovarian (OVCAR, A2780, A2780DDP, SK-OV-3); colorectal (HT29, HS700T, DLD-2, Colo201, HCT116); pancreatic (PANC-1); non-small-cell lung (SK-MES-1, MV-522, HOP-62, SKLU, SKNH-SH, A549) and small-cell lung (NCI-H522) cancer cell lines along with Burkitt's lymphoma (Raji, Daudi, Ramos); and acute lymphoblastic leukaemia (CCRF-CEM) cell lines were successively tested in this study. All cell lines but MCF7-VHK4, MCF7-V4B, A2780 and A2780DDP were from American Tissue Culture Collection (ATCC, Rockville, MD, USA). Mutant p53 MCF7-VHK4 cells (substitution at codon 179 resulting in a mutation from histidine to glutamine) derived from parental wild p53 MCF-7 (oestrogen and

*© 1999 Cancer Research Campaign* 

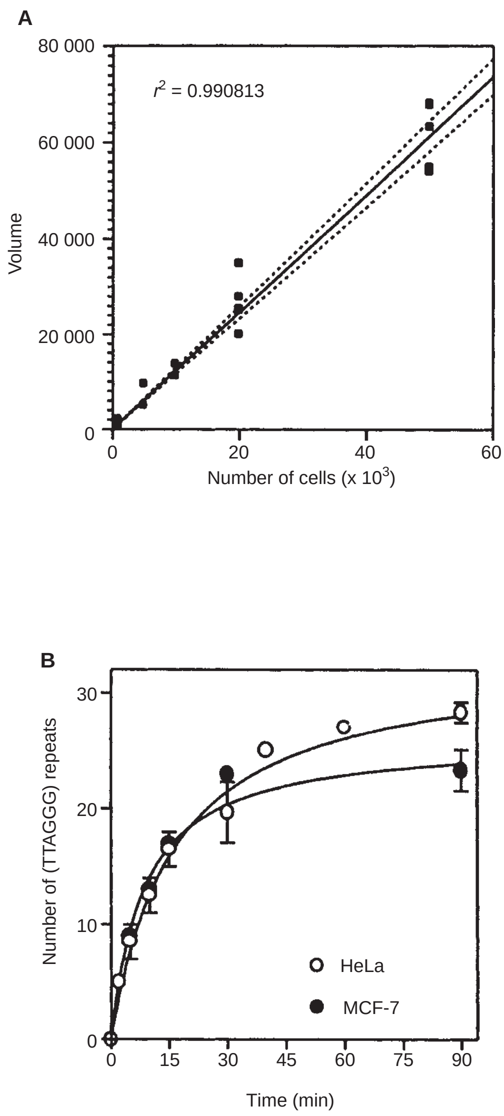

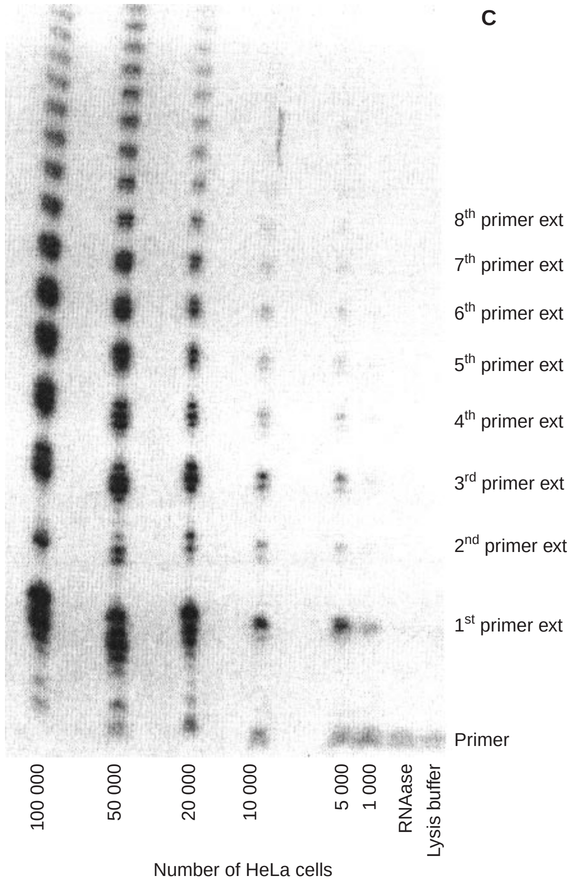

**Figure 2** Telomerase activity in HeLa cell detergent extracts using a newly designed biotinylated-primer extension telomerase assay. The assay allows the direct measurement of the enzyme activity. (**A**) The activity level (volume) was calculated by summation of integrated areas of the telomerase ladder after background subtraction. The signal resulting from this assay is proportional to the number of cells (i.e. the protein concentration in the extract) and allows for the detection of a minimum of 1000 HeLa cells. (**B**, **C**) The reduction of the radioactive background on the autoradiogram allows for a clear visualization of the first primer extensions and the direct visualization telomerase activity allows the determination of the number of repeats added to the primer with time by telomerase progesterone receptors positive) after transfection of the cDNA derived from a mutated lung cancer cell line using a CMV promoter for p53 expression, control MCF7-V4B cells containing only the vector, and MCF7mdr were obtained from Dr R Elledge (UTHSC, San Antonio, TX, USA). Parental A2780 and A2780DDP (resistant to cisplatin) were obtained from Dr K Scanlon (City of Hope, Duarte, CA, USA).

Normal cell were used as negative control. Normal human RPMI 7666 lymphoblasts (ATCC) and MRC-9 lung fibroblast cell lines (ATCC), and non-immortalized fibroblasts, endothelial and epithelial cells from breast and prostate (all from Clonetic, San Diego, CA, USA) were also tested in this assay. Normal and cancer cells were grown in appropriate media in a humidified 37°C incubator with 5% carbon dioxide.

### Effects of cytotoxic drugs

The telomerase activity was evaluated in MCF7 cells cultured in the presence of 5%, and 15% fetal bovine serum (FBS) or exposed to antiproliferative agents including 4OH-cyclophosphamide (Sigma, St Louis, MO, USA), doxorubicin (Sigma), cisplatin (Sigma), and $TMPyP_4$ (Gift from Laurence Hurley, Department of Pharmacy at the University of Texas, Austin, TX, USA). To ensure that at least 10 million viable cells would be available for making telomerase extracts after exposure to cytotoxic drugs, twenty million cells were seeded in a T-150 flask and grown 24 h before the addition of drugs. The cells were exposed to the drug for 2–24 h, then washed and cultured in a drug-free media. The viability was determined on a haemocytometer with Trypan blue. Ten million control and drug-treated viable cells were serially aliquoted for analyses at day 4, day 8 and day 15 after the drug removal.

In vivo experiments were performed in Harlan (*nu/nu*) athymic mice (Sprague, Indianapolis, IN, USA) bearing transplantable MX-1 human mammary tumour xenografts. MX-1 is a fast-growing human tumour commonly used for the preclinical evaluation of anticancer drugs (Plowman et al, 1997). MX-1 breast cancer xenografts were previously shown to be predictive of human response to new anticancer drugs (Plowman et al, 1997). Care of the mice was in accordance with institutional and Federal

*© 1999 Cancer Research Campaign*

guidelines. Mice were treated with a single intraperitoneal injection of cisplatin at the dose of 9 mg kg$^{-1}$, known to induce a significant tumour response. At least five mice were used per experimental group. Tumours were measured every 2 days and mice were sacrificed when the tumour size was 50% of those from the control group. Tumours from control and cisplatin-treated groups were minced and repeatedly passed through metal sieves with 40-µm mesh (EC Apparatus, St Petersburg, FL, USA) to obtain a single-cell suspension. Specimens were washed twice in McCoy's 5A medium containing 5% horse serum (Sigma), 10% heat-inactivated fetal calf serum (FCS; Hyclone, Logan, UT, USA), 2 mM sodium pyruvate, 2 mM glutamine, 90 U ml$^{-1}$ penicillin, 90 µg ml$^{-1}$ streptomycin and 35 µg ml$^{-1}$ L-serine (all Gibco). The viability of cells was determined on a haemocytometer with Trypan blue.

### Preparation of telomerase extracts

Only viable cells were used for telomerase experiments. Control and drug-treated cells were processed side by side to ensure accurate comparisons. For all samples, an aliquot containing $10^7$ viable cells was used for the telomerase extract. In all cases, cell samples were cryopreserved at –196°C in liquid phase nitrogen, then placed on ice (+4°C) just before telomerase extraction. Samples containing suspensions of $10^7$ cells as a single-cell suspension were washed once in phosphate-buffered saline (PBS) (400 µl) and pelleted at 10 000 ***g*** for 1 min at 4°C. Cells were subsequently resuspended in 400 µl of ice-cold washing buffer containing 10 mM HEPES-KOH (pH 7.5), 1.5 mM magnesium chloride ($MgCl_2$), 10 mM potassium chloride (KCl) and 1 mM dithiothreitol (DTT), then pelleted again at 10 000 ***g*** for 1 min at 4°C. Washed cells were resuspended in 200 µl ($10^6$ cells per 20 µl) of ice-cold lysis buffer containing 10 mM Tris–HCl (pH 7.5), 1 mM $MgCl_2$, 1 mM EGTA, 0.1 mM phenylmethylsulphonyl fluoride, 5 mM β-mercaptoethanol, 1 mM DTT, 0.5% 3-[(3-cholamidopropyl)-dimethyl-ammonio]-1-propanesulphonate (CHAPS), 10% glycerol and 40 UI ml$^{-1}$ RNAase inhibitor (Promega, Madison, WI, USA). The suspension was incubated on ice for 30 min. This usually permitted the lysis of 90–100% of cells. The lysate was then transferred to polyallomer tubes (Beckman, Fullerton, CA, USA) and spun at 100 000 ***g*** for 1 h at 4°C in a tabletop ultracentrifuge (Beckman). The supernatant was removed and the protein concentration in the extracts was determined by the Bradford assay (BioRad, Hercules, CA, USA) before all telomerase experiments. The protein concentration ranged usually between 4 and 10 µg µl$^{-1}$. When necessary (protein concentration < 4 µg µl$^{-1}$), telomerase extracts were concentrated using Microcon Microfilter 30 (Amicron, Beverly, MA, USA) to reach a final protein concentration ranging from 4 to 10 µg µl$^{-1}$. Then, the extract was quickly stored at –80°C.

### Telomerase assay

Telomerase activity was measured with our newly developed non-amplified assay (Figure 1). An aliquot of the extract containing 10–15 µg of protein (2–5 µl of CHAPS extract) was used for each telomerase assay. The telomerase extract was assayed in 20 µl of reaction mixture containing 2 mM dATP, 2 mM dTTP, 0.6 µM α-$^{32}$P-dGTP and 1 µM 5′-biotinylated $(TTAGGG)_3$ primer, in the presence of 1.428 µl of 14× telomerase reaction buffer containing 0.7 M Tris KOAC, 14 mM $MgCl_2$, 70 mM spermidine and 14 mM β-mercaptoethanol. The solution was incubated for 1 h at 37°C for telomerase extension of the primer. The reaction was terminated by the addition of streptavidin-coated magnetic beads followed by a magnetic separation of the bound primers. Telomerase extension products bonded to the beads were washed several times with a solution of 1 M sodium chloride (NaCl), in 10 mM Tris–HCl solution (pH 7.5) to eliminate non-incorporated α-$^{32}$P-dGTP. The washing process was facilitated by utilization of magnetic stands (Dynal, New York, NY, USA). Subsequently, extended primers were separated from the beads by incubation for 30 minutes in a solution of 5.0 M guanidine–HCl at 90°C. Following ethanol precipitation, telomerase reaction products were analysed by electrophoresis in an 8% polyacrylamide denaturing gel. Gels were dried on filter paper and developed by autoradiography on an ultrasensitive Biomax MS film (Kodak, Rochester, NY, USA).

In the presence of processive telomerase activity, various numbers of hexameric repeats are added to the 18-bp primer, yielding a 6-bp incremental DNA ladder. Extracts from cells not containing telomerase do not extend the primer. Enzyme activity was quantified by autoradiograph followed by scanning on a Molecular Dynamics PhosphoImager (Sunnyvale, CA, USA) and the density of each band of the repeat ladder was determined using ImageQuant software (Molecular Dynamics, Sunnyvale, CA, USA).

HeLa cells have been shown to have a high and reproducible telomerase activity in the conventional telomerase assay, and therefore that cell line was used as a positive control and for normalization of the level of telomerase activity in our assay. Total activity was expressed as a percentage of that in HeLa cell extract (positive control) after normalizing for protein content. Experiments in breast cancer cell lines were performed at least in duplicate. A sample was considered positive for telomerase activity if the signal was abrogated by pre-incubation with RNAase I (Worthington Biochemical Corp., Freehold, NJ, USA). Extracts were considered negative if no extension was detected after 7 days of exposure to ultrasensitive Biomax-MS films (Kodak) using two Biomax-MS intensifying screens (Kodak).

### Telomere length analysis

Genomic DNA was prepared by incubating cell pellets in 2 ml of lysis buffer (0.1 M NaCl, 10 mM Tris (pH 8.0), 25 mM EDTA (pH 8.0), 0.5% sodium dodecyl sulphate (SDS), and 0.1 mg ml$^{-1}$ proteinase K for 1 h at 37°C, followed by phenol extraction and ethanol precipitation. DNA pellets were resuspended in a solution containing 10 mM Tris (pH 7.6), 1 mM EDTA (pH 8.0) and restriction digests were done with Sau 3A1 in southern blots with hybridization to a TTAGGG telomeric probe. Approximately 10 µg of digested DNA per lane was loaded onto a 0.8% agarose gel. Southern blotting was performed by standard methods, and blots were hybridized with α-$^{32}$P-labelled oligonucleotide $(TTAGGG)_3$ at 42°C for 18 h. Prior to restriction digestion, a portion of each DNA sample was electrophoresed to verify its integrity. Telomere lengths were measured with an ultrascan densitometer, with the center of the peak taken as the mean telomeric restriction fragment length. For several data sets, telomere lengths were also calculated as previously described following scanning of the southern blot autoradiograms with a digital scanner. Experiments were performed at least in duplicate.

© 1999 Cancer Research Campaign 

### RESULTS

### Telomerase activity in conventional primer extension telomerase assay

To confirm the linearity of our biotinylated-primer extension telomerase assay (Figure 1), we initially performed a dilution experiment with HeLa cells. We observed a linear relationship between the number of cells in the extract and the CHAPS-extract protein concentration in the telomerase reaction (data not shown). Telomerase activity was readily detectable in samples containing at least 500–1000 HeLa cells.

The level of telomerase was proportional to the protein concentration, i.e. to the number of cells in the extract (Figure 2A). As shown in Figure 2C, the reduction of the radioactive background on the autoradiogram allows for a clear visualization of the first primer extension and the number of TTAGGG-repeats added by telomerase with time (Figure 2B). Assaying the same sample at three different occasions, led to 4% variation of the signal demonstrating a good reproducibility of this method.

As expected, the telomerase activity was abolished in RNAase I pretreated samples (Figure 2C) as well as in samples heated at 95°C for 10 min prior to the telomerase reaction (data not shown). Using the procedure described above, the protein concentration in cancer cell extracts typically ranged from 6 to 8 μg $\mu l^{-1}$. A HeLa cell extract that was aliquoted, was quickly frozen (–80°C), and was stable for at least five freeze–thaw cycles and for more than 6 months which ensured comparison between experiments.

Using this method, we observed a 3-band pattern for each telomerase extension (Figure 2C). This was never reported before using other methods for the measurement of telomerase activity. However, conventional methods and the TRAP assay for telomerase measurement either do not allow the direct visualization of the banding pattern due to either a lack of sensitivity due to a high background (conventional assay) or do not measure the signal directly resulting from the incorporation of nucleotides due to the amplification process. Our method for telomerase measurement that used a long polyacrylamide denaturing gel similar to those used for sequencing gels allows the discrimination of each incorporated radiolabelled nucleotide. Since there is no amplification,

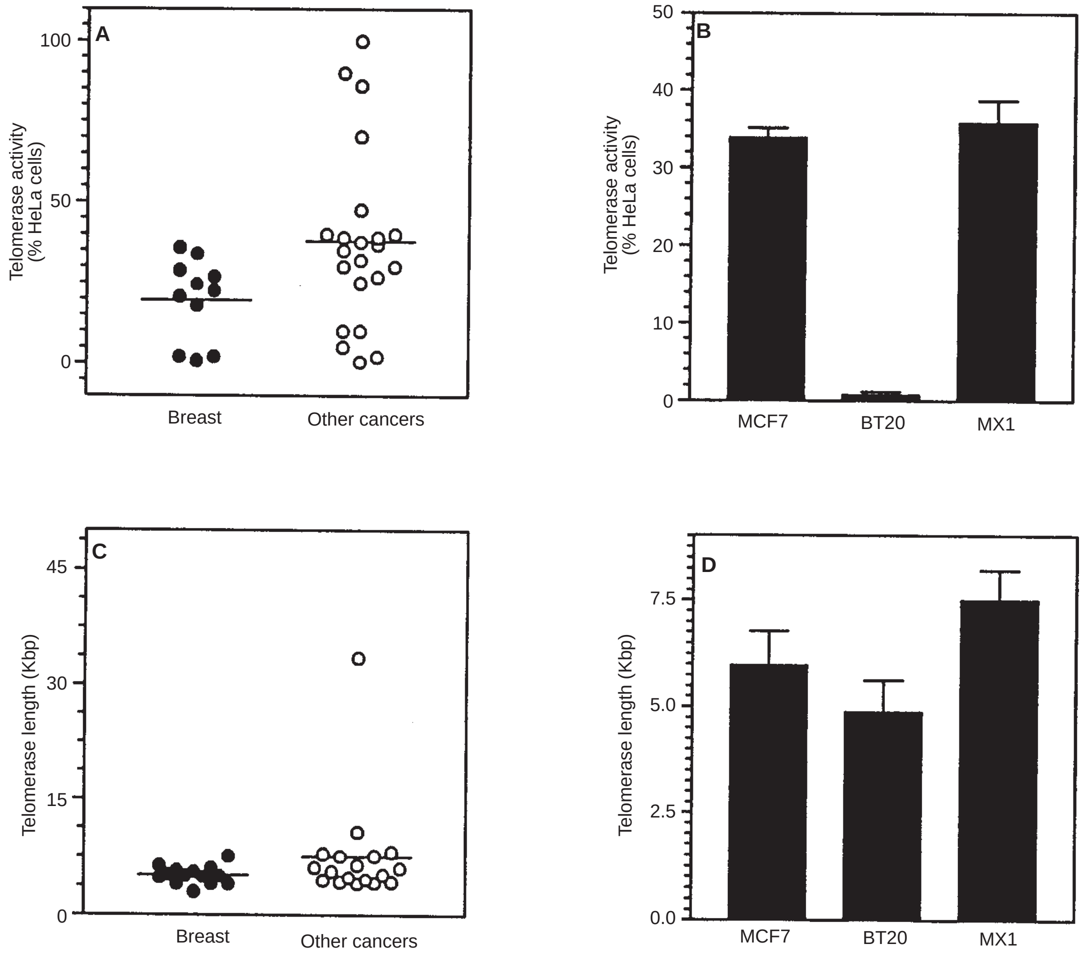

**Figure 3** Telomerase activity and telomere lengths in breast cancer cells. The biotinylated-primer extension telomerase assay was performed using extracts containing 100 000 cell-equivalents. The telomerase activity in cell lines was compared to that of HeLa cells (% of HeLa cell activity). The telomerase activity varied markedly with cell lines, but no significant difference in telomerase was observed between breast cancer and other tumour types (**A**). Breast cancer cell lines MCF7 and MX1 showed high telomerase activity while no telomerase activity was detected in BT20 (**B**). The telomere length in breast cancer cell line was similar to that of a panel of cell lines from a large variety of tumour types (**C**). There was no significant correlation between telomere length and telomerase activity. For example, BT20 that shows low telomerase activity with our method had maintained telomere length suggesting that mechanisms other than telomerase can extend the telomeric region of DNA in this particular cell line (**D**)

 *© 1999 Cancer Research Campaign*

the observed banding directly results from the direct incorporation of $^{32}$P-radiolabelled dGTP on the biotin-$(TTAGGG)_3$ primer. Therefore, this 3-band pattern suggests that telomerase pauses after each $^{32}$P-radiolabelled dGTP incorporation.

### Telomerase activity in human breast cancer cell lines

Comparing telomerase signals in a variety of 39 human cancer cell lines to that of HeLa cells, we observed that the level of telomerase varied markedly from cell line to cell line (31.8 ± 24.9%, range: 0–100, Figure 3A). No significant difference in telomerase activity was observed in individual cell lines taken at different passages under similar standard growth conditions (data not shown). Similarly, the doubling time and tumorigenicity of individual cell lines did not correlate with telomerase activity (data not shown). With our method, only three out of the 39 cell lines studied (7.7%, BT20, SKLU and ZR75-1) did not show any telomerase activity. Normal fibroblasts, normal endothelial and normal epithelial cells at early passages did not show processive telomerase activity (data not shown). In this study, the average telomerase activity in breast cancer cell lines was 19.6 ± 12.7% (range: 0–35.75) versus 37.8 ± 27.4% (range: 0–100) in other types of human cancer cell lines. We showed that MCF7 breast cancer cells expressed a high level of telomerase activity (33.8 ± 3.4%, range: 30–36.6) compared to other breast cancer cell lines (Figure 3A, B). Interestingly, the level of telomerase in MCF7 was very stable from one experiment to another. We further showed that oestrogen-independent, oestrogen-dependent, doxorubicin-resistant and p53-mutated MCF7 cells expressed similar levels of telomerase activity (data not shown). Interestingly, using our method, we did not detect any significant telomerase activity in BT20 breast cancer cells in several experiments (Figure 3B). This cell line was further tested using the TRAP assay, which allow the detection of telomerase activity in this cell line. This suggests that a low level of telomerase is present in this cell line. However, using our assay we can assume that the level of telomerase in BT20 is at least 1000 times lower than in MCF7.

### Telomere lengths in human breast cancer cell lines

We showed that telomere lengths varied with cell line (average: 6.24 ± 6.16 Kbp). No significant difference in telomere length was observed in individual cell lines taken at different passages under similar standard growth conditions. The average telomere length in human breast cancer was 5.1 ± 1.1 Kb versus 7.5 ± 6.7 Kb in other tumour types (Figure 3C). In the MCF7 cell line, which showed processive telomerase activity, the telomere length was 5.2 ± 1.1 kbp (range: 3.8–6.63, Figure 3D). In the BT20 breast cell line, which did not show processive telomerase activity, the average telomere length was 4.3 ± 0.03 Kb (range: 4.2–4.3). No correlation between the telomere lengths and the levels of telomerase was observed suggesting that high processivity of the telomerase enzyme is not essential for telomere maintenance. Moreover, while the level of telomerase activity was very low in BT20 cells, the telomere length was comparable to that of other breast cancer cells with higher telomerase activity (Figure 3D). This suggests that a mechanism other than telomerase expression may be responsible for telomere elongation in this particular cell line.

### Effects of drugs on telomerase activity in vitro

The effects of cytotoxic drugs were tested in MCF7 cells. The growth of MCF7 cells in the presence of drugs was determined prior to the telomerase experiment to identify concentrations inducing about 25%, 50% and 75% growth inhibition. The cells that were exposed to concentrations of 4OH-cyclophosphamide below 0.5 μM for 1 h ($IC_{50}$) and those exposed to concentrations of doxorubicin below 0.01 μg ml$^{-1}$ for 1 h ($IC_{50}$) had no significant decrease in telomerase activity (data not shown). Higher concentrations often induced dramatic cytotoxicity that did not allow us to maintain cells in culture. We observed 48.8%, 50.8% and 81.4% decrease of MCF7 cell growth after a 4-day exposure to 0.1, 1.0 and 10.0 μM cisplatin respectively (Figure 4A). The effects of cisplatin on the cell growth was transient with a quick recovery by day 8 after exposure. As shown in Figure 4A, the telomerase activity was significantly reduced at day 4 in cells exposed to these concentrations of cisplatin. Telomerase inhibition closely followed the cell growth with recovery at day 8 after cisplatin exposure. In a cell-free system, cisplatin did not inhibit telomerase suggesting indirect effects in cells (data not shown). Because cisplatin is known to bind to guanine-rich regions of DNA, we subsequently attempted to determine whether it could affect telomere stability. In a cell-free system, we observed that incubation of the telomeric primer for more than 12 h with cisplatin was associated with primer degradation (data not shown). However, in cultured cells, attempts to maintain MCF7 cell growth in the presence of cisplatin at concentrations ≥ 0.1 μM were associated with dramatic cytotoxicity. Protracted exposure to concentrations of cisplatin < 0.1 μM did not significantly reduce telomerase activity and was not associated with telomere shortening (data not shown).

The cytotoxic effects of $TMPyP_4$ on telomerase activity was investigated in MCF7 cells in culture. $TMPyP_4$ is a fluorescent compound that rapidly accumulates into the cells and concentrates into the nucleus few hours after incubation. No significant antiproliferative effects were observed at concentration ranging from 1 to 50 μM and a 30% growth inhibition was observed at 100 μM. As shown in Figure 4C, $TMPyP_4$ reduced telomerase activity at concentration ranging from 1 to 100 μM in vitro. However, no significant reduction of the telomere length was observed after 15 days of exposure to $TMPyP_4$ (data no shown).

### In vivo model for the study of telomerase activity

We observed that the level of telomerase and telomere length of MCF7 human tumour xenografts in mice was identical to that observed in cells in culture (data not shown). Moreover, MX1 breast cancer xenografts commonly used for the preclinical evaluation of anticancer drugs showed a high level of telomerase corresponding to 35.75 ± 7.15 (range: 28.6–42.9%) of that of HeLa cells. The average telomere length in MX1 cells was 7.5 ± 0.5 Kb (range: 7–8). Extracts from mice leucocytes, gut, liver and skin did not express telomerase activity in our assay (data not shown).

We subsequently evaluated the effects of cisplatin on telomerase activity in MX1 breast cancer xenografts in mice. As shown in Figure 4B, 9 mg kg$^{-1}$ cisplatin was very effective in inducing tumour shrinkage in MX1 xenografts. To evaluate the effects of cisplatin on telomerase activity in xenografts, we sacrificed mice immediately before and 5 days after cisplatin treatment. At the time of sacrifice both group had about the same tumour volume. Figure 4B shows that a decrease in telomerase activity can be observed 5 days after treatment with cisplatin in MX1 breast cancer xenografts in mice. Few mice that did not show response to therapy had no decrease in telomerase activity as compared to the control (data not shown).

*© 1999 Cancer Research Campaign*

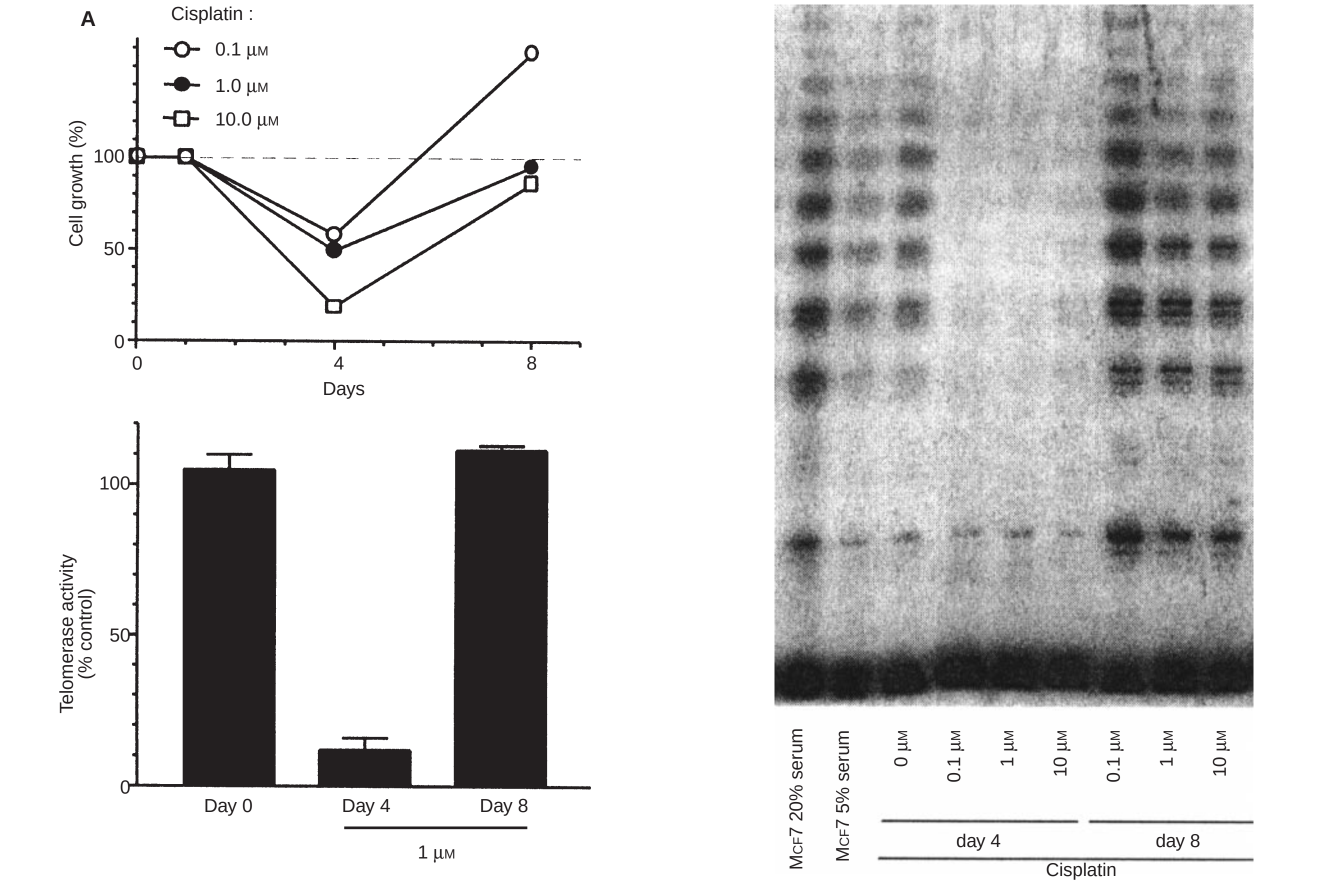

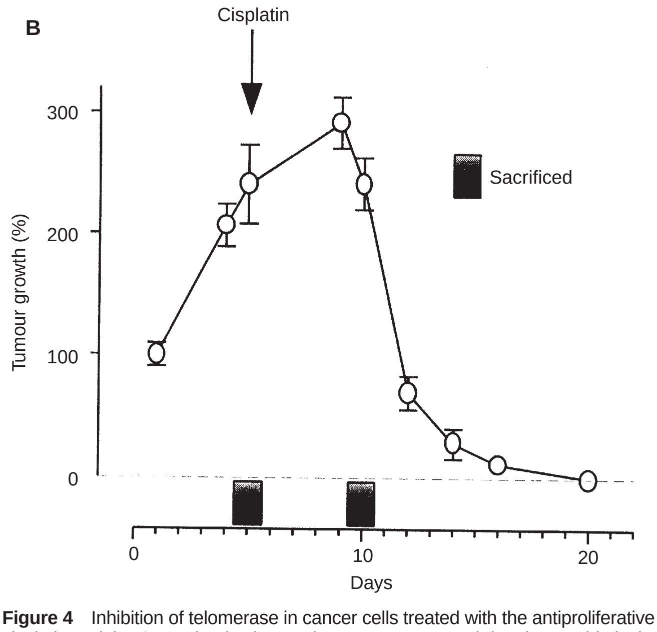

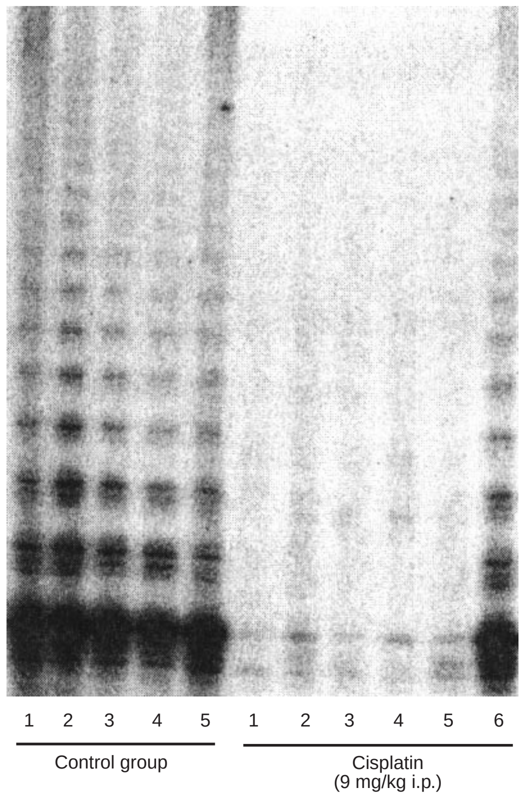

**Figure 4** Inhibition of telomerase in cancer cells treated with the antiproliferative drug cisplatin and the G-quadruplex interacting agent TMPyP4. (**A**) Using our biotinylated primer extension assay, we showed that we were able to detect and quantify the variations of telomerase activity in MCF7 cells exposed to several concentrations of cisplatin in vitro. We observed a transient reduction of telomerase activity in MCF7 cells exposed to cisplatin in vitro. (**B**) Athymic mice bearing MX1 xenografts were treated with 9 mg $kg^{-1}$ intraperitoneal cisplatin. This dose of cisplatin induces a complete regression of tumours in mice. A group of mice was sacrificed before treatment with cisplatin and another group 5 days after treatment. The volume of tumour in both groups was similar. Telomerase activity in treated tumours was decreased as compared to the control group. (**C**) Using the same assay, we quantified telomerase activity in vitro in MCF7 cells using a G-quadruplex interacting agent $TMPyP_4$ that induces potent telomerase inhibition at concentrations ranging from 10 to 100 µM

*© 1999 Cancer Research Campaign*

C

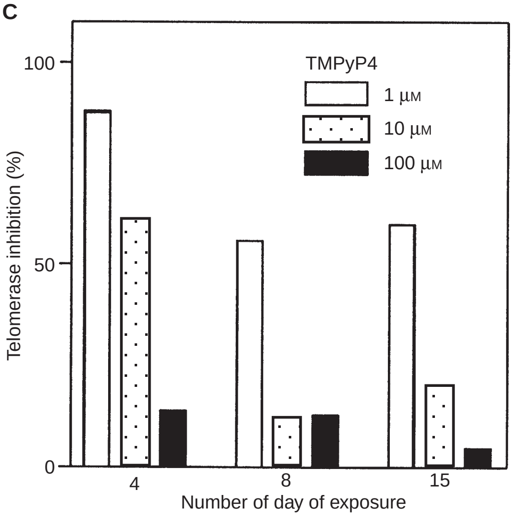

**Figure 4** cont

### DISCUSSION

In this study we applied a new method for investigating telomerase activity in breast cancer cells in culture. Our method allows the direct measurement of telomerase activity through incorporation of a labelled nucleotide. The use of a 5′-biotinylated primer allows for the capture of telomerase-extended primers using streptavidine-coated magnetic beads. Then, using magnets that allow the separation of extended primers, residual $^{32}$P-dGTP can be washed out and the signal resulting from the incorporation of labelled dGTP onto the primer becomes distinguishable from the background. The utilization of excess of streptavidine-coated magnetic beads allows for the recovery of the biotinylated primers extended by telomerase. Based on our data, the TRAP-assay which detects telomerase activity in fewer than 10 cells over a million, is undoubtedly more sensitive than our method, which requires 500–1000 cells to detect telomerase activity. However, the TRAP assay has several limitation for whom wanting to quantify telomerase inhibition. Those limitations have been described by Kim et al (1994) in the original publication then re-evaluated by several other authors (Holt et al, 1996) leading to subsequent improvement of the TRAP-method. Major limitations of the TRAP assay are related to quantification and PCR-derived artifacts. There have been several attempts to improve the quantification of telomerase activity using internal controls, using standard telomerase-positive cell lines, and using a quantification control in the commercially available TRAP assays. When we started to investigate the effects of telomerase inhibitors, the quantification of telomerase activity was our primary concern. Problems potentially resulting from the interaction of our compounds with the TS-primer, the CX primer and the *Taq* polymerase were likely to result in false-positive or false-negative results. Moreover, false-positive results that might result from primer dimer artifacts present in the TRAP assay might be favoured by DNA-interacting agent that stabilized unspecific primer dimer formations. In addition, synthetic primers used as substrates for telomerase in PCR-methods do not behave like (TTAGGG) sequences when exposed to some alkylating or intercalating agents designed to bind G-quartets such as $TMPyP_4$. Since, in our lab, we were investigating G-quadruplex interacting agents, it was highly preferable to consistently use the same method for telomerase measurement from the biochemical reactions to the in vivo assay. We observed that our method provides a significant advantage over current PCR-based method by the fact that it is a one-step reaction that showed on the gel the direct incorporation of nucleotides in the telomeric primer and by displaying elongation products that unlike the TRAP assay does represent the processivity of the enzyme. In addition, our method uses the human TTAGGG-sequence as a template for telomerase reaction and might be considered for investigating G-quadruplex interacting compounds. Of interest, our method prevents a direct interaction between the tested compound and the TS-primer, the CX-primer and the *Taq* polymerase. In addition, this method avoids primer dimer artifacts.

However, limitation of our method should also be underlined. As mentioned above, the sensitivity of our method is undoubtedly lower than that of the TRAP assay (Kim et al, 1994; Holt et al, 1996) but higher than that of other conventional primer extension protocols currently used (Shay et al, 1994). Therefore, due to the lack of sensitivity, this method is unsuitable for the measurement of telomerase using small tissue specimens that contain a limited number of cells. In addition, this method requires a larger amount of $^{32}$P dGTP as compared to the TRAP assay, require basic knowledge in molecular biology, and need more practice than the TRAP assay that is now available as a kit. Therefore, our method could not be recommended for routine clinical detection of telomerase in specimens from patients, but instead could be used for laboratory experiments in which the experimental setting allows use of a large number of cells.

Several previous reports have indicated that telomerase is expressed in breast cancer and may play an important role in carcinogenesis and tumour progression (Shay et al, 1993*b*, 1995*b*; Hiyama et al, 1996; Bednarek et al, 1997; Tsao et al, 1997). Therefore, inhibition of telomerase may represent a new strategy against this particularly frequent malignancy. However, few models have been specifically developed to study the biological effects of those inhibitors in human cancer cells. Chadeneau et al (1995) have reported telomerase activity using the TRAP assay in mammary tumour cells from transgenic mice overexpressing the *neu* gene. However, Prowse et al (Prowse et al, 1993; Prowse and Greider, 1995) have shown that, while human telomerase can processively synthesize long TTAGGG repeats in vitro, the mouse telomerase appears to be much less processive. Those differences in processivity may be related to differences in telomerase enzyme protein structure and function and therefore suggest that mouse telomerase may not be suitable for the study of compounds specifically designed to target human telomerase (Raymond et al, 1996). Moreover, based on previously published data (Chadeneau et al, 1995) and in our experience, the telomere lengths in transgenic mouse tumour cells are usually 10–100-fold longer than that of human cancer cells. Therefore, a certain number of cell divisions, which may exceed the average life time of mice, may be required before telomere shortening becomes manifest phenotypically in transgenic mice. For these reasons transgenic models may not be suitable for preclinical evaluation of new compounds designed to target human telomerase and telomeres in advanced cancer models.

In this study, we showed that telomerase activity varies markedly between different human cancer cell lines. However, there was no correlation between the activity of the enzyme and the telomere length. Authors have reported that immortalized cells can maintain their telomeres by other, but still unidentified, mechanisms that do not involve telomerase (Bryan et al, 1995). We

© 1999 Cancer Research Campaign 

showed that telomerase-negative immortalized cells appear infrequently (less than 8% in our study). We showed that breast cancer cell lines are attractive models for future evaluation of compounds that target telomerase and telomeres. For example, the high level of telomerase in MCF7 and MX1 cells and the relatively short telomere lengths represent reasonable parameters for the testing of new compounds. MX1 is a fast-growing human tumour commonly used for the preclinical evaluation of anticancer drugs but, unfortunately, does not grow in vitro (Plowman et al, 1997). MCF7 cancer cells grow in vitro but is a slow-growing tumour in vivo. Moreover, MCF7 express CEA biological marker and offer a large variety of derived counterparts such as doxorubicin-resistant, hormone-refractory and p53-mutated cell lines with similar telomerase activity that can be subsequently evaluated to closely determine in which situation new compounds may have optimal activity. Together, MX1 and MCF7 provide an interesting human breast cancer model for the testing of potential telomere–telomerase interactive agents in vitro and in vivo.

Predictive mathematical simulations indicate that after inhibition of telomerase a relatively long period of time corresponding to about 20–30 cell divisions would be necessary for telomerase inhibitors to induce a dramatic telomere shortening and chromosomal instability (Vojta and Barret, 1995). Such a number of cell divisions is theoretically sufficient to increase the tumour mass by 1000 times in patients and is certainly sufficient to kill the host (Harley et al, 1992, 1994; Harley and Villeponteau, 1995). This lag time is incompatible with life for a patient with a rapidly growing human breast cancer. Therefore, the clinical development of telomerase inhibitors will need to integrate this lag time period into any new drug development strategy (Raymond et al, 1996). Such a strategy may incorporate conventional chemotherapy in order to reduce the tumour size before the utilization of telomerase inhibitors in patients with minimal residual disease after debulking induction chemotherapy (Sharma et al, 1997). To determine whether anticancer agents which can be used to initially reduce the tumour can also modulate telomerase activity, we evaluated the effects of some classic agents against MCF7 breast cancer cells. We investigated the effects of doxorubicin and 4OH-cyclophosphamide, which are considered major anticancer drugs in the treatment of patients with advanced breast cancers, on telomerase activity and telomere lengths. Since recent reports also indicate that cisplatin may be an effective inhibitor of telomerase (Burger et al, 1997), we subsequently tested the effects of cisplatin on telomerase activity in breast cancer cells in vitro and in vivo. We observed that various concentrations of cisplatin and high concentration of cyclophosphamide and doxorubicin can decrease the telomerase activity. Our results are consistent with other reports indicating that cytotoxic effects of anticancer drugs can be monitored by measuring telomerase activity (Burger et al, 1997; Faraoni et al, 1997). High concentrations of cytotoxic drugs are likely to affect telomerase expression by a non-specific inhibition of DNA, RNA and/or protein synthesis. Interestingly, we observed that relatively low concentrations of cisplatin, which transiently inhibit the cell growth, were also able to transiently down-regulate telomerase. We showed that this inhibition was not due to direct interaction between cisplatin and the telomerase enzyme. However, telomerase inhibition induced by cisplatin was only transient and despite the potential of cisplatin to bind G-rich DNA region, long-term exposure to low concentrations of cisplatin was not associated with telomere shortening. Unlike HeLa cells (Ishibashi et al, 1998), telomeres of MCF7 cells do not shorten and degrade after cisplatin exposures. Recent reports indicate that cisplatin indirectly effects telomerase in cancer cells through the interaction with a telomerase-RNA encoding gene region (Burger et al, 1997). Combined, these results suggest that telomerase can be temporarily down-regulated in cancer cells after high-dose chemotherapy. To investigate whether telomerase can be effectively down-regulated in tumours, we monitored the telomerase activity in mice bearing MX1 breast cancer xenografts after treatment with active doses of cisplatin. We observed that the tumour shrinkage induced by cisplatin in this model was associated with a significant decrease of the telomerase activity. These results suggest that classical chemotherapy may not only induce tumour shrinkage in human cancer but may also transiently inhibit telomerase. In addition, we showed that a compound with mild antiproliferative effects such as $TMPyP_4$, specifically designed to interact with telomeres and telomerase in vitro (Sun et al, 1997; Wheelhouse et al, 1998), can induce telomerase inhibition in cultured cells. However, since no significant telomere shortening was observed after a 14-day exposure, it is likely that those compounds will need very prolonged exposure and/or co-exposition to other telomere interactive agents to shorten telomeres. Our results suggest that advanced breast cancer may be a valid preclinical and clinical tumour for testing of new drugs that target telomerase. Moreover, our data support the utilization of a high-dose debulking chemotherapy prior to treatment with telomerase inhibitors in breast cancer to accelerate telomerase inhibition and overcome the lag time problem expected with telomerase inhibitors.

In summary, we have implemented a new method utilizing a non-amplified telomerase assay to measure the level of telomerase in breast cancer cells. Using this method, we showed that breast cancer is a reasonable human tumour model for the study of telomerase inhibitors that are being developed (Izbicka et al, 1997). Furthermore, our data suggest that pretreatment with conventional chemotherapeutic agents may be incorporated in the setting of preclinical and clinical trials with new telomerase inhibitors to both rapidly shrink the tumour and reduce the telomerase activity in advanced human breast cancers.

### ACKNOWLEDGEMENTS

The authors thank Dr Sandrine Faivre, Gregory Hannibal, Alice Goodwin and Laida Garcia for their assistance in preparing the manuscript for publication. This study was supported in part by National Institutes of Health/National Cancer Institute grant # 67760 for the National Cooperative Drug Discovery Group (NCDDG), Sanofi Research, and the Cancer Therapy and Research Foundation of South Texas, San Antonio, TX. Dr. Eric Raymond was the recipient of a grant from the Association pour la Recherche contre le Cancer (ARC) and a grant from l’Assistance Publique des Hôpitaux, France. Dr. Sunil Sharma was supported by an NCI Clinical Oncology Research Cancer Development Award K12CA01723. This study was presented in part during the 89th Annual Meeting of The American Association for Cancer Research, New Orleans, LA, USA.

### REFERENCES

Allsopp RC and Harley CB (1995) Evidence for a critical telomere length in senescent human fibroblasts. *Exp Cell Res* **219**: 130–136

Bednarek AK, Sahin A, Brenner AJ, Johnston DA and Aldaz CM (1997) Analysis of telomerase activity in breast cancer: positive detection at the in situ breast carcinoma stage. *Clin C Res* **3**: 11–16

 © 1999 Cancer Research Campaign

Blackburn EH (1991) Structure and function of telomeres. *Nature* **350**: 569–573
Bryan TM, Englezou A, Gupta J, Bacchetti S and Reddel RR (1995) Telomere elongation in immortal human cells without detectable telomerase activity. *EMBO J* **14**: 4240–4248
Burger AM, Double JA and Newell DR (1997) Inhibition of telomerase activity by cisplatin in human testicular cancer cells. *Eur J Cancer* **33**: 638–644
Chadeneau C, Siegel P, Harley CB, Muller WJ and Bacchetti S (1995) Telomerase activity in normal and malignant murine tissues. *Oncogene* **11**: 893–898
Faraoni I, Turriziani M, Masci G, De Vecchis L, Shay JW, Bonmassar E and Graziani G (1997) Decline in telomerase activity as a measure of tumor cell killing by antineoplastic agents in vitro. *Clin Cancer Res* **3**: 579–585
Fisher B (1996) Personal contributions to progress in breast cancer research and treatment. *Sem Oncol* **23**: 414–427
Gilley D and Blackburn EH (1994) Lack of telomere shortening during senescence in *Paramecium*. *Proc Natl Acad Sci USA* **91**: 1955–1958
Greider CW (1993) Telomerase and telomere-length regulation: lessons from small eukaryotes to mammals. *Cold Spring Harbor Symposia on Quantitative Biology* **58**: 719–723
Greider CW (1994) Mammalian telomere dynamics: healing, fragmentation shortening and stabilization. *Curr Opin Genet & Development* **4**: 203–211
Harley CB, Kim NW, Prowse KR, Weinrich SL, Hirsch KS, West MD, Bacchetti S, Hirte HW, Counter CM, Greider CW et al (1994) Telomerase, cell immortality, and cancer. *Cold Spring Harb Symp Quant Biol* **59**: 307–315
Harley CB, Vaziri H, Counter CM and Allsopp RC (1992) The telomere hypothesis of cellular aging. *Exp Gerontol* **27**: 375–382
Harley CB and Villeponteau B (1995) Telomeres and telomerase in aging and cancer. *Curr Opin Genet Dev* **5**: 249–255
Harrington L, McPhail T, Mar V, Zhou W, Oulton R, EST program A, Bass MB, Arruda I and Robinson MO (1997) A mammalian telomerase-associated protein. *Science* **275**: 973–977
Healy KC (1995) Telomere dynamics and telomerase activation in tumor progression: prospects for prognosis and therapy. *Oncol Res* **7**: 121–130
Hiyama E, Gollahon L, Kataoka T, Kuroi K, Yokoyama T, Gazdar AF, Hiyama K, Piatyszek MA and Shay JW (1996) Telomerase activity in human breast tumors. *J Natl Cancer Inst* **88**: 116–122
Holt SE, Norton JC, Wright WE and Shay JW (1996) Comparison of the telomeric repeat amplification protocol (TRAP) to the new TRAP-eze telomerase detection kit. *Methods Cell Sci* **18**: 237–248
Ishibashi T and Lippard SJ (1998) Telomere loss in cells treated with cisplatin. *Proc Natl Acad Sci USA* **95**: 4219–4223
Izbicka E, Wheelhouse RT, Raymond E, Davidson KK, Lawrence RA, Sun D, Windle BE, Hurley LH and Won Hoff DD (1999) Effects of cationic porphyrins as G-quadruplex interactive agents in human tumor cells. *Cancer Res 59*: 639–644
Kim NW, Piatyszek MA, Prowse KR, Harley CB, West MD, Ho PL, Coviello GM, Wright WE, Weinrich SL and Shay JW (1994) Specific association of human telomerase activity with immortal cells and cancer. *Science* **266**: 2011–2015
Levy MZ, Allsopp RC, Futcher AB, Greider CW and Harley CB (1992) Telomere end-replication problem and cell aging. *J Mol Biol* **225**: 951–960
Marcand S, Gilson E and Shore D (1997) A protein-counting mechanism for telomere length regulation in yeast. *Science* **275**: 986–990
Piccart M, Raymond E, Aapro M, Eisenhauer EA and Cvitkovic E (1995) Cytotoxic agents with activity in breast cancer patients previously exposed to anthracyclines: current status and future prospects. *Eur J Cancer* **31A**: 1–10
Plowman J, Dykes DJ, Hollingshead M, Simpson-Herren L and Alley MC (1997) Human tumor xenograft models in NCI drug development. In: *Anticancer Drug Development Guide*, Teicher BA (ed), pp. 101–125. Humana Press: Totowa, NJ
Price CM (1993) Telomere structure and function. *Ind J Biochem Biophys* **30**: 77–82
Prowse KR and Greider CW (1995) Developmental and tissue-specific regulation of mouse telomerase and telomere length. *Proc Natl Acad Sci USA* **92**: 4818–4822
Prowse KR, Avilion AA and Greider CW (1993) Identification of a non-processive telomerase activity from mouse cells. *Proc Natl Acad Sci USA* **90**: 1493–1497
Raymond E, Sun D, Chen S-F, Windle B and Von Hoff DD (1996) Agents that target telomerase and telomeres. *Curr Opin Biotechnol* **7**: 583–591
Sharma S, Raymond E, Soda H, Sun D, Hilsenbeck SG, Sharma A, Izbicka E, Windle B and Von Hoff DD (1997) Preclinical and clinical strategies for development of telomerase and telomere inhibitors. *Ann Oncol* **8**: 1063–1074
Shay JW, Van Der Haegen BA, Ying Y and Wright WE (1993*a*) The frequency of immortalization of human fibroblasts and mammary epithelial cells transfected with SV40 large T-antigen. *Exp Cell Res* **209**: 45–52
Shay JW, Wright WE and Werbin H (1993*b*) Toward a molecular understanding of human breast cancer: a hypothesis. *Breast Cancer Res Treat* **25**: 83–94
Shay JW, Brasiskyte D, Ouellette M, Piatyszek MA, Werbin H, Ying Y and Wright WE (1994) Analysis of telomerase and telomeres. In: *Methods in Molecular Genetics, Vol. 5, Part C, Genes and Chromosome Analysis*. Adolph KW (ed), pp. 263–280. Academic Press: New York
Shay JW, Piatyszek MA, Word RA, Gazdar AF, Wright WE, Kim NW, Weinrich SL, Prowse KR, Harley CB, Hiyama E, Hiyama K, Mehle C, Roos G, Sommerfeld HJ, Meeker AK and Coffey DS (1995*a*) You have not heard the end of it: telomeres, time, telomerase and tumors (Meeting abstract). *Proc Annu Meet Am Assoc Cancer Res* **36**, 673
Shay JW, Tomlinson G, Pityszek MA and Gollahon LS (1995*b*) Spontaneous in vitro immortalization of breast epithelial cells from a patient with Li–Fraumeni syndrome. *Mol Cell Biol* **15**: 42–432
Sun D, Chen S-F and Von Hoff D (1996) A sensitive and rapid assay method of human telomerase using dynabeads. In: *9th NCI-EORTC Symposium on New Drugs in Cancer Therapy*, Lobbezoo MW (ed), pp. 71 (A240). Kluwer: Amsterdam
Sun D, Thompson B, Cathers BE, Salazar M, Kerwin SM, Trent JO, Jenkins TL, Neidle S and Hurley LH (1997) Inhibition of human telomerase by G-Quadruplex-interactive compounds. *J Med Chem* **40**: 2113–2116
Thompson B, Salazar M, Sun D, Jenkins T, Neidle S and Hurley L (1996) Telomerase inhibition by G-quartet interactive compounds. In: *9th NCI-EORTC Symposium on New Drugs in Cancer Therapy*, Lobbezoo MW (ed), pp. 71 (A243). Kluwer: Amsterdam
Tsao J-I, Zhao Y, Lukas J, Yang X, Shah A, Press M and Shibata D (1997) Telomerase activity in normal and neoplastic breast. *Clin Cancer Res* **3**: 627–631
Vojta PJ and Barret JC (1995) Genetic analysis of cellular senescence. *Biochem Biophys Acta* **1242**: 29–41
Wheelhouse RT, Sun D, Han H, Han FX and Hurley LH (1998) Cationic porphyrins as telomerase inhibitors: the interaction of tetra (*N*-methyl-4-pyridyl)porphyrine with quadruplex DNA. *J Am Chem Soc* **120**: 3261–3262

*© 1999 Cancer Research Campaign*
